# Experiences Using Media Health Claims to Teach Evidence-Based Practice to Healthcare Students: A Mixed Methods Study

**DOI:** 10.12688/f1000research.146648.2

**Published:** 2024-09-17

**Authors:** Ida-Kristin Orjasaeter Elvsaas, Hilde Tinderholt Myrhaug, Lisa Garnweidner-Holme, Jürgen Kasper, Astrid Dahlgren, Marianne Molin

**Affiliations:** 1 Department of Nursing and Health Promotion, Oslo Metropolitan University, Oslo, Oslo, 0130, Norway; 2 School of Health Sciences, Kristiania University College, Oslo, Oslo, 0107, Norway

**Keywords:** Evidence-Based Practice, Evidence-Based Healthcare, Critical Thinking, Health Literacy, Health Education, Professional Education, Mixed Method Design

## Abstract

**Background:**

A multifaceted and interactive teaching approach is recommended for achieving proficiency in evidence-based practice, with critical thinking considered vital for connecting theory and practice. In this context, we advocate the strategic use of health claims in the media to promote critical thinking, complemented by a blended learning approach and a group exam.

**Method:**

We conducted a convergent mixed methods study, including a cross-sectional survey with structured and open-ended questions as well as focus group interviews, at Oslo Metropolitan University, during the 2020-2021 academic year. Participants were bachelor’s students in healthcare. We employed a blended learning approach, combining digital learning resources, teaching in critical assessment of health claims and evidence-based practice, and a concurrent group exam assignment centered around media health claims. The outcome measures included students’ experiences integrating health claims into evidence-based practice teaching and their experiences with teaching approaches and the group exam.

**Results:**

Out of 465 participants, 136 (29.2%) responded to the structured questions in the survey. In response to the open-ended questions within the survey, 109 (80.1%) of the respondents shared positive experiences about the course, while 98 (72%) suggested improvements. Additionally, 25 students participated in focus group interviews. Synthesizing the results, we found that students viewed the inclusion of health claim assessment as a useful entry point for learning evidence-based practice. In addition, the students identified both the blended learning design and the group exam as contributors to a positive perception of learning outcomes from the course.

**Conclusions:**

Based on student feedback, integrating critical reflection on media health claims into evidence-based practice education, alongside a blended learning approach and a group exam, may be beneficial in teaching evidence-based practice to bachelor’s healthcare students. However, further rigorous study designs are needed to objectively assess the effect of the course on learning outcomes.

**Registration:**

DOI 10.5281/zenodo.6985449

## Background

Evidence-based practice (EBP) is essential for enhancing healthcare quality and safety. ^1^^–^^3^ Educational programs in healthcare professions should incorporate EBP to ensure that graduates can deliver high-quality, evidence-based care. ^4^^,^^5^ In Norway, higher education institutions are legally obligated to equip health and welfare science students with EBP competencies. ^6^

While the core components of EBP are generally agreed upon, ^3^^,^^4^^,^^7^ successful teaching and learning in EBP require interactive and multifaceted approaches integrated with clinical practice. ^5^ These teaching strategies should incorporate various assessment methods to gauge students’ progress and comprehension. ^5^ In addition, cultivating critical thinking skills is considered essential for bridging the gap between theory and practice, allowing students to understand the relevance of evidence in their future professional roles. ^8^ To address this issue, we propose using health claims in the media to promote critical thinking and provide an entry point for EBP for university healthcare students ^9^ in the early stages of their education and lack of clinical practice experience. Health claims found in news articles, social media posts, and advertisements are easily identifiable and hold relevance to students’ daily lives, ^9^ as well as their future professional practice when faced with health claims from patients and caregivers. ^10^^,^^11^ Previous studies have shown this approach to be successful at teaching the public to think critically about health claims. ^12^^,^^13^ Consequently, there is potential for applying this strategy in professional education, fostering similar skills during training, and enhancing comprehension of the practical implementation of EBP.

### News stories in the media as a learning resource in EBP

During the academic year 2020 to 2021, Oslo Metropolitan University (OsloMet) adopted a blended learning approach to deliver a newly developed three-week course on evidence-based practice in healthcare (hereafter called the EBHC course). The EBHC course was designed to align with the common learning outcomes for bachelor’s students in health and social services education, thereby becoming a mandatory course for first- or second-year students at the Faculty of Health Sciences. Despite the novelty of the EBHC course at our university, it was underpinned by the foundational elements of EBP instruction based on the Norwegian adaptation of the Critical Appraisal Skills Program (CASP) model. ^14^ In addition, the course incorporated a module with elements from a previous learning design called “Behind the Headlines”, ^9^ serving as a stepping-stone to EBP. The “Behind the Headlines” section builds on the Informed Health Choices (IHC) Key Concept Framework and focuses on assessing treatment claims. ^15^ In this context, treatment claims refer to actions or products used to maintain or improve health. Generic examples of such claims include statements as, “This product can help you lose weight,” “This exercise can reduce stress and anxiety” and “This treatment can help manage chronic pain.” The initial implementation of the EBHC course encompassed four study disciplines: bioengineering, physiotherapy, social education, and occupational therapy.

### Objective

This study aimed to explore healthcare students’ experiences during the first year of implementing our EBHC course. The outcome measures encompassed their experiences integrating health claims into EBP teaching, as well as their experiences with teaching approaches, and the group exam.

## Methods

We employed a convergent mixed methods research design, ^16^ which included a cross-sectional survey and focus group (FG) interviews. The convergent design is a single-phase approach that involves the collection of quantitative and qualitative data, followed by separate analyses, culminating in an integrated analysis. ^16^^,^^17^ The combination of structured and open-ended questions in the questionnaire, along with FG interviews, was used to comprehensively understand participants’ experiences. Our goal was to extract fresh insights that surpass the results obtained from separate analyses of the quantitative and qualitative data. ^17^

The reporting of the study was guided by the Strengthening the Reporting of Observational Studies in Epidemiology (STROBE) Statement, ^18^ the Consolidated Criteria for Reporting Qualitative Research (COREQ), ^19^ and the Mixed Methods Appraisal Tool (MMAT), Version 2018. ^20^

### Ethics

This study evaluates healthcare education. Although OsloMet does not have an Ethics Review Board to approve projects, researchers are committed to adhering to ethical standards following applicable national regulations, institutional guidelines, and the principles of the Declaration of Helsinki (as revised in 2013). ^21^

The data in the study were collected anonymously, without personal or indirect personal information, or health and illness data. Therefore, according to the guidelines of the Norwegian Agency for Shared Services in Education and Research, no approval from them was necessary. Before participating in the study, all participants provided written informed consent for the collection and use of data for research purposes.

### Setting and participants

We included healthcare students who participated in the EBHC course from 2020 to 2021. The eligible participants were 465 first- and second-year bachelor students enrolled in bioengineering, physiotherapy, social education, and occupational therapy programs at the Faculty of Health Sciences at OsloMet. Separate courses were conducted for each study program.

To collect survey data, announcements were posted on “Canvas,” the learning management system utilized by OsloMet, toward the end of the courses. A reminder was posted 4 to 7 days later. Participation in the survey was voluntary and anonymous, although respondents who completed the survey had the opportunity to enter a lucky draw to win NOK 500 gift cards. However, to participate in the draw, respondents needed to express their interest by emailing one of the lecturers. There was no connection between the submitted answers and participants’ email addresses to ensure anonymity.

Toward the end of the EBHC courses, an invitation to participate in the FG interviews was issued through “Canvas,” with participants selected based on convenience sampling. Each participant in the FG interviews was offered a gift card worth NOK 300.

### The EBHC course

**Figure 1.**
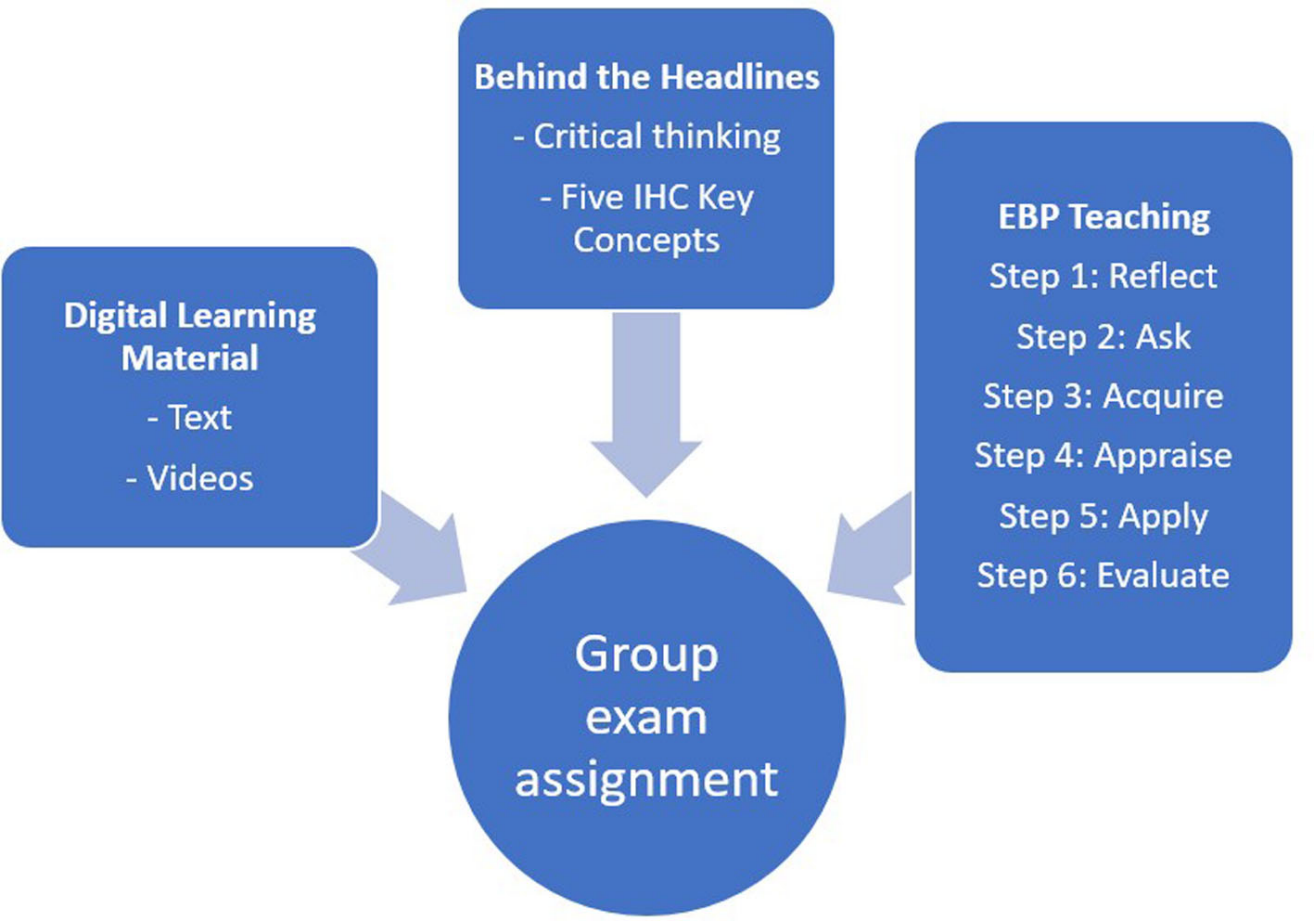
The components of the EBHC course.

The three-week EBHC course ( Figure 1) comprised digital learning materials, lectures, and student activities in “ Behind the Headlines” and EBP, ^4^ with concurrent student collaboration on a group exam assignment. The three-week EBHC course ( Figure 1) comprised digital learning materials, lectures, and student activities in “ Behind the Headlines” and EBP, ^4^ with concurrent student collaboration on a group exam assignment. The course was structured for online teaching to accommodate the large student body. Health claims were consistently incorporated throughout its structure to achieve the learning outcomes. A blended learning strategy, which included elements from a flipped classroom approach, was implemented for teaching. A group exam centered around media health claims was used to assess the learning outcomes. In-class learning activities included eight half-day or full-day seminars (e.g., help desk, critical appraisal seminars, and seminars to present preliminary group exam work and peer feedback). Time was also allocated for individual study (in line with the flipped classroom approach). For the course generic timetable, see *Extended data.* ^22^

At the beginning of the course and before each new topic, students were encouraged to use digital learning materials covering the EBHC curriculum. These materials were accessed via the learning management system (“Canvas”) and consisted of textual content as well as links to videos and other multimedia resources.

In the “Behind the Headlines” module, students were asked to critically reflect on the reliability of health claims made by the media regarding treatment effects. This was achieved through an assessment of the basis for these health claims using five key concepts ( Table 1) adopted from the Informed Health Choices (IHC) Key Concept Framework. ^15^ These five key concepts were previously chosen as essential elements of the “Behind the Headlines” learning design. This design, detailed in Oxman et al., ^9^ serves as the foundation for the EBHC module titled “Behind the Headlines.”

**Table 1.** Overview of IHC Key Concepts introduced in the Behind the Headlines module.

1.	Beliefs alone about how treatment work may not be relevant.
2.	An outcome may be associated with a treatment but not caused by it.
3.	The results of one study considered in isolation can be misleading.
4.	Small studies may be misleading.
5.	Fair comparisons of treatment in animals or highly selected groups of people may not be relevant.

The students were taught EBP in six sequential steps ( Figure 1). This involved reflecting on information needs (Step 1) based on the evaluation of one or more health claims from the media, formulating questions using the PICO framework (Step 2), conducting literature searches (Step 3), critically appraising research studies (Step 4), gaining insight into implementing (Step 5), and evaluating (Step 6) EBP in clinical settings.

*Learning activity structure*

**Figure 2.**
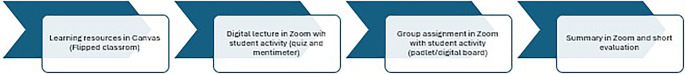
The “Behind the Headlines” module in the EBHC course.

Our EBHC course aimed to provide a uniform learning experience for all healthcare students. Therefore, most seminars, including those in the “Behind the Headlines” module depicted in Figure 2, followed a similar structure. They began with a plenary session lasting one to three sessions of 45 minutes each, featuring a presentation from the lecturer. During this plenary session, students were encouraged to clarify misunderstandings or highlight particularly challenging areas. Subsequently, students worked in groups of approximately six to seven participants, with assignments provided in Canvas. These group sessions lasted about one to three hours, depending on the topic. During the group work, one or two lecturers would visit the groups in their breakout rooms for clarification and discussion. Each seminar culminated in a forty-five-minute plenary session, during which volunteer groups presented their findings. This session also provided an opportunity for these groups to engage in discussions about challenging aspects of the assignments with other groups in attendance at the seminar.

To ensure a standardized learning experience for students, we adopted a consistent approach across different fields of study. This involved using the same learning materials provided in Canvas and employing teaching methods in EBP based on the Norwegian adaptation of the CASP model. ^14^ All lecturers were familiar with the teaching material, and there was a collective agreement on the content of the presentations. Furthermore, most of the teaching modules were delivered by the same lecturers across all courses.

*Exam assignment*

In the course’s first week, a group exam assignment was distributed, requiring students to critically assess the basis of one (out of several) provided health claims from the media using the five IHC Key Concepts ( Table 1). Real-world examples of media health claims utilized include: “Acupuncture is most effective for back pain,” “Creatine enhances performance and muscle volume,” “MMR vaccine potentially causes autism,” and “Laser treatment improves neck conditions.” This critical assessment process also prompted them to reflect and identify information needs, followed by formulating a research question using the PICO framework and conducting targeted literature searches. Additionally, they were required to provide a relevant checklist for appraising the methodological quality of the selected research article. Finally, the students discussed whether the health claim made in the news article was supported by the selected research article and explored the potential relevance of these findings for patients or the Norwegian health service.

### Data collection and analysis

Data were gathered individually for each discipline but analyzed collectively across these fields. This methodological approach was chosen to align with the study’s objective of exploring healthcare students’ experiences with the EBHC course.

*Evaluation survey*

The data were collected anonymously through a questionnaire created using nettskjema.no, a survey solution developed and hosted by the University of Oslo. The questionnaire ^22^ included several sections, starting with background information such as gender, age, and study program. Furthermore, there was a course evaluation section featuring Likert scale questions and open-ended questions.

Two of the Likert scale questions in the questionnaire focused on course information (“ *To what extent did the information provided by the course managers effectively serve its purpose in informing you about the course?*”) and overall learning outcomes (“ *Please rate your learning outcomes in the course*”). The respondents were asked to rate these items from one (very good) to four (not good at all). The learning outcomes were further explored through other learning strategies used (detailed in Table 3), where students indicated their level of agreement on a scale from 1 (completely agree) to 6 (completely disagree), with an additional option for “do not know.” In addition, there was a section for participants to provide other comments.

In the open-ended questions, students were allowed to provide feedback on the EBHC course by answering the following questions: “ *Please share any positive aspects you found in the course, such as the content, teaching methods, organization, work style, assessment, etc.*” and “ *Do you have any suggestions for improving the course, such as the content, teaching methods, organization, work style, assessment, etc.?*”.

### Statistical methods

Descriptive statistics were used to analyze the respondents’ characteristics, and frequencies were employed to analyze the responses to each Likert scale evaluation question. In addition, the responses related to the detailed learning outcomes were collapsed and categorized into “agree” (ratings of 1, 2, and 3 on the 6-point scale), “disagree” (ratings of 4, 5, and 6 on the 6-point scale) and “do not know”. The data were analyzed using IBM SPSS Statistics version 27 software. ^23^

### Qualitative analysis

The answers to the open-ended questions were analyzed using the systematic text condensation strategy as follows: 1) obtaining an overall impression; 2) identifying and organizing meaningful units; 3) condensing the information; and 4) synthesizing the condensed material. ^24^ The analytical steps were carried out independently by two researchers. Subsequently, the researchers engaged in discussions to achieve a common understanding of the findings.

*Focus group interviews*

We utilized Rosenbaum’s honeycomb framework ^25^ as the basis for developing the FG interview guide. ^22^ This framework encompasses various problem aspects and differentiates between multiple facets of user experience. The FG interviews were conducted via Zoom following OsloMet’s privacy policy for Zoom interviews ( https://ansatt.oslomet.no/en/rutine-zoom-forskningsintervjuer) and lasted for approximately one hour. The FG interviews were not audio recorded. One researcher was the moderator while two other researchers took notes during the interviews. All the participating researchers then cross-checked these notes. Any discrepancies or differences of opinion among the researchers were resolved through consensus. The student participants did not provide feedback on the notes.

The notes obtained from the FG interviews were analyzed using the systematic text condensation strategy, ^24^ following the same analytical approach applied to the responses to the open-ended questions in the evaluation survey.

*Mixed methods approach*

To facilitate the synthesis of results from the structured and open-ended questions in the survey and focus group interviews, we initially analyzed similarities and differences in the datasets, considering their respective purposes and the responses they generated. Furthermore, to gain insights beyond what can be derived from the separate analysis of the quantitative and qualitative datasets, we synthesized and presented the findings in a joint display, ^17^ visually integrating the data.

### Reflexivity

In qualitative research, which also encompasses a mixed-methods approach, researchers play an integral role in the research process, and our prior experiences, assumptions, and beliefs will therefore inevitably shape its trajectory and outcomes. ^26^ The authors of the study have diverse backgrounds, and experience in conducting systematic reviews, EBP teaching and research, and clinical and academic areas. The team collaborated to ensure investigator triangulation.

Two of the co-authors (HTM and MM) actively participated in the development of the course and the teaching process and conducted the FG interviews. However, the remaining co-authors were not involved in either the teaching or the FG interviews. To address potential biases in the analysis of the qualitative data, two researchers (HTM and IKOE) independently conducted the data analysis before reaching a shared understanding. This methodological approach, together with the extensive use of quotations from open-ended questions and FG interviews, was used to strengthen the credibility of the study findings. ^27^

## Results

To ensure transparency, the underlying quantitative and qualitative data are made publicly available. ^22^

### Quantitative results from the evaluation survey

Our survey included 136 out of 465 eligible participants, representing a response rate of 29.2% ( Table 2). There were no missing values. ^22^ Most of the study participants were women, accounting for 79.4% of the sample, and a substantial proportion were younger than the age of 26 (78.0%). All the various fields of study were represented, with the highest representation from physiotherapy (32.4%) and the lowest from occupational therapy (19.1%).

**Table 2.** Study participants in the evaluation survey.

	Evaluation survey (n = 136)
**Female,** n (%)	108 (79.4)
**Age (year),** n (%)	
≤ 20	62 (45.6)
21-25	44 (32.4)
26-30	14 (10.3)
31-35	2 (1.5)
≥36	14 (10.3)
**Study program,** n (%)	
Bioengineering	34 (25.0)
Physiotherapy	44 (32.4)
Social Education	32 (23.5)
Occupational therapy	26 (19.1)

A total of 75.7% of the students rated the course information as either “good” or “very good,” while 90.5% reported a positive perception of their overall learning outcome, indicating that it was either “good” or “very good”.

The overall learning outcome was further investigated through eight questions related to different educational methods implemented in the EBHC course. When analyzing these subcategories, students exhibited the highest satisfaction within the collaborative exam group, with 50.4% completely agreeing with the statement “ *I have experienced positive learning outcomes from working in the exam group.*” The subcategories related to experienced learning outcomes for other resources or teaching methods displayed variability. However, upon categorizing the results of the subcategories of learning outcomes as “agree,” “disagree,” and “do not know,” it became evident that these learning outcome measures leaned toward agreement, as indicated in Table 3.

**Table 3.** The proportion of responses to the learning outcome subcategories.

I have experienced positive learning outcomes from:	Agree, %	Disagree, %	Do not know, %
*Digital learning resources in general*	70.6	28.6	0.8
*Digital learning resources related to “Behind the Headlines”*	70.9	26.2	2.9
*Digital learning resource from the Western Norway University of Applied Sciences*	68.0	27.6	4.4
*Lectures*	74.3	25.7	0
*Problem-solving seminars with teachers*	73.5	22.7	3.8
*Working in the exam group*	78.0	20.3	1.7
*Reading syllabus on their own*	66.5	29.1	4.4
*Combination of campus and digital teaching*	48.7	20.0	31.3

### Results from the open-ended questions in the evaluation survey

In total, 80.1% (109/136) of the students answered what they regarded as positive about the course and 72.1% (98/136) suggested improvements. See *Extended data* ^22^ for the distribution of responses to the open-ended questions in the evaluation survey.

*Positive feedback about the course*

Analysis of the positive feedback about the course was divided into topics about (1) digital learning resources, (2) student learning activities, (3) group exam collaboration, and (4) the usefulness of the course. Additional quotes supporting the topics can be found in *Extended data.* ^22^

The availability of comprehensive digital learning resources both before and after the lessons was appreciated by many. For instance, as one student wrote, “ *The organization of self-learning through Canvas etc … was fantastic and easy to follow along.*”

The students held a positive perception of the student learning activities, expressing the lecturers’ proficiency in facilitating diverse learning activities such as quizzes, group discussions, seminar presentations, and peer reviews. A student commented, “ *Exciting content, committed lecturers, fun with collaborative tasks/discussion in plenary.*”

The incorporation of the group exam early in the course and thus ample time for reflection received positive feedback. A student noted, “ *You got so much more out of the course when you worked in groups. Then you could discuss with each other and generally hear what others think. You could be good together.*”

Additionally, the students expressed appreciation for the acquisition of critical assessment skills regarding health claims in the media, familiarity with the evidence pyramid, and the ability to critically appraise scientific research papers, considering these components relevant to their future professional endeavors. A student wrote, “ *I think being able to be critical of health claims on the internet was incredibly good.*” Furthermore, another student wrote, “ *Everything about this course has been educational and useful. It is very relevant for future assignments and bachelor’s thesis.*”

*Suggestions for improvement*

The analysis of the open questions about suggestions for improvements was distributed among the topics of (1) digital learning resources, (2) education, and (3) the group exam. Additional quotes supporting the topics can be found in *Extended data*. ^22^

Some students expressed concerns about the organization of the learning management system (“Canvas”) and the presence of two similar platforms from different universities, one affiliated with OsloMet in English and the other with the Western Norway University of Applied Sciences in Norwegian. A student who articulated this concern serves as an example, writing *“Difficult to find the right information, as there were several Canvas rooms and a bit “messy” with information in both English and Norwegian.”* As a suggestion for improvement, a student wrote, “ *As for the organization inside Canvas, I think this was very good when I first understood it, but there should perhaps have been a little clearer information about how to find and use Canvas, this learning platform is new and unfamiliar to many so early in the studies.”*

Concerns were raised about the efficacy of breakout rooms, as many students either did not actively participate or were unprepared, resulting in a lack of meaningful discussion. For instance, a student wrote, *“Many digital lectures had almost only breakout rooms, with very little academic content. I felt there was no point in discussing something I didn’t know with someone who also didn’t know [the academic content].”* This perspective was echoed by others, for example, a student who wrote, *“There were a lot of breakout rooms/seminars which were a bit of a waste. That there was unnecessary time spent in breakout rooms.”*

Although the group exam was generally successful, some concerns arose regarding the possibility of free riders and the appropriate approach to managing such situations. A student commented, *“The group project method – having to do the exam in a group – will always be challenging when some contribute a lot and are very engaged and interested, while others are checked out and unmotivated.”* A possible solution to the free rider problem was expressed by a student, writing *“In the case of further online group work, I think [there should be] a requirement to log attendance so that everyone contributes and that it is not difficult for the students to report if someone wandered off.”*

### Results from the Focus Group interviews

Out of 29 interested students, 25 from the various programs participated in the FG interviews. The first FG interview included five bioengineering students, the second involved eight physiotherapy students, the third comprised five social education students, and the fourth consisted of seven occupational therapy students. Details regarding participation and drop-outs are provided in *Extended data,* ^22^ although no reasons were given for the dropouts.

The analysis of the FG interviews covered the following topics: (1) digital learning resources, (2) course comprehension, (3) the relevance of health claims, (4) group collaboration, and (5) the course’s usefulness. Several pertinent quotations corroborating the topics are available in *Extended data.* ^22^

The students acknowledged the presence of diverse information within the learning management system ("Canvas"), as exemplified by one comment from a student, *"[…] good [learning] resources in Canvas.*" However, some initially perceived its layout to be disorganized. As they became more familiar with the system, they found navigation to be effortless. One student expressed that “ *[It was] easy to find [the information] once you became familiar [with Canvas].”*

At the outset of the course, most students showed limited familiarity with the concept of evidence-based practice and the course content. However, they appreciated the course’s well-structured progression and the clarity of the learning materials, making the content easily understandable, aided by the logical steps in EBP teaching. One student expressed, “ *Well structured, easy to follow. Everything was clear.*”

Health claims, introduced through the “Behind the Headlines” module, were acknowledged as a useful entry point for EBP, as expressed by one student, “ *Behind the headlines and EBP are connected. EBP is sort of next step …*”. This could be attributed to their direct relevance to everyday experiences. One student expressed “ *I think it would have been much duller if you had removed [the health claims]. That’s how [the course] becomes relevant for us.*” Another student further emphasized the significance of health claims for their future profession, stating, “ *You can get patients who base their knowledge on [health claims]. [We] must be able to refute them sensitively.*”

Several students emphasized the importance of group collaboration as a catalyst for fostering critical reflection. A student expressed, “ *It is important to have a group when we are going to be critical, several points of view emerge.*” Another student echoed a similar sentiment, expressing an appreciation for the collaborative learning approach, “ *I liked that it was group work. It makes it easier to learn when you are in a team. Easier to discuss.*”

The students perceived the course as highly beneficial, serving multiple purposes. These included enhancing their ability to write their bachelor’s thesis, critically assessing health claims encountered in their everyday lives, and preparing themselves for their forthcoming roles as updated healthcare practitioners, informed by best practices. One student expressed, “ *I have already started searching the Health Library (“Helsebiblioteket”) to prepare for practice, [I have] never done [that] before.*”

All the students articulated that the course provided them with the necessary skills to critically assess media claims. One student expressed that “ *Being critical of sources is useful. [Behind the Headlines] was like an ABC.*” Moreover, the students reported the acquisition of skills in locating trustworthy research and the ability to autonomously appraise its validity. One student highlighted that “ *[I have] learned to be critical of research - learned good ways to be critical - a proper recipe.*” Furthermore, the students acknowledged the universal significance of this knowledge, emphasizing its potential benefits for a broader audience.

### Results from the mixed methods synthesis

Our datasets served distinct purposes and provided diverse insights. The structured questions in the survey primarily evaluated learning outcomes, lacking inquiries about, for example, the usefulness of “Behind the Headlines” for understanding the concept of EBP. Students provided open-ended responses in the survey concerning content, teaching methods, organization, work style, and the exam. The primary concerns centered around how teaching methods, organization, and work style prepared them for the exam, with some offering feedback on the practical applicability of course content. The FG interviews provided an in-depth understanding of students’ experiences with the EBHC course, including the integration of health claims as a fundamental aspect of the educational process.

We identified three main outcomes addressing our study objective through the synthesis of results obtained from our datasets, as presented in Table 4. First, we found that critical reflection on health claims introduced through the “Behind the Headlines” module was a useful entry point for EBP. Second, the blended learning design had a positive influence on the students’ perceived learning outcomes. Finally, the collaborative exam assignment promoted both critical reflection and positively impacted perceived learning outcomes.

**Table 4.** Joint display of the quantitative and qualitative data synthesis *.

Main findings	Quantitative survey	Open-ended questions	FG interviews
**Critical reflection on health claims as an introduction to EBP**	N/A	***Positive feedback***	“ *Behind the Headlines and EBP are connected. EBP is sort of next step …*”
		“ *I think being able to be critical of health claims on the internet was incredibly good.*”	
		***Suggestions for improvement***	“ *I think it would have been much duller if you had removed [the health claims]. That’s how [the course] becomes relevant for us.*”
		N/A	
			“ *You can get patients who base their knowledge on [health claims]. [We] must be able to refute them sensitively.*”
			*“Health claims are most (compared to scientific literature) recognizable in everyday life. It is important to learn how to deal with this”* (From Suppl. 8)
**Blended learning design on perceived learning outcomes**	Most students reported positive learning outcomes from the digital learning materials: (1) in general, 70.6%, (2) to “Behind the Headlines,” 70.9%, (3) from the Western Norway University of Applied Sciences, 68.0%	***Positive feedback***	*"[*… *] good [learning] resources in Canvas.*"
		“ *The organization of self-learning through Canvas etc … was fantastic and easy to follow along.*”	
		“ *Exciting content, committed lecturers, fun with collaborative tasks/discussion in plenary.*”	“ *[It was] easy to find [the information] once you became familiar [with Canvas].”*
		“ *The structure of the teaching, where you first prepare, then lecture and then work with assignments [was positive].”* (From Suppl. 5)	“ *Well structured, easy to follow. Everything was clear.*”
	Most students (74.3%) reported positive learning outcomes from lectures	“ *Nice assignments and exciting with active lectures. It forced me to pay more attention and I learned more.*” (From Suppl. 5)	
	Most students (73.5%) reported positive learning outcomes from problem-solving seminars with teachers	***Suggestions for improvement***	
		“ *As for the organization inside Canvas, I think this was very good when I first understood it, […].”*	
		*“Many digital lectures had almost only breakout rooms, with very little academic content. […].”*	
	Most students (66.5%) reported positive learning outcomes from reading the syllabus on their own	*“There were a lot of breakout rooms/seminars which were a bit of a waste. That there was unnecessary time spent in breakout rooms.”*	
**The collaborative exam for critical reflection and perceived learning outcomes**	Most students (78.0%) reported positive learning outcomes from working in the exam group	***Positive feedback***	*“It is important to have a group when we are going to be critical, several points of view emerge.*"
		“ *You got so much more out of the course when you worked in groups. Then you could discuss with each other and generally hear what others think. You could be good together.*”	
		” *[Positive to] way of working, working in groups and getting different opinions on issues is always educational and fun*” (From Suppl. 5)	" *I liked that it was group work. It makes it easier to learn when you are in a team. Easier to discuss.*"
		“ *I got the most out of the exam assignment. Nice to present the task to other groups. This led to greater insight into the entire assignment and constructive feedback from other students.*” (From Suppl. 5)	*“Nice to work in groups. [Awarding!]”* (From Suppl. 8)
		***Suggestions for improvement***	
		*“The group project method – having to do the exam in a group – will always be challenging when some contribute a lot and are very engaged and interested, while others are checked out and unmotivated.”*	
		*“In the case of further online group work, I think [there should be] a requirement to log attendance so that everyone contributes and that it is not difficult for the students to report if someone wandered off.”*	

* Most of the quotes are directly translated from Norwegian to English using Google Translate. Therefore, the wording and sentence structure may be somewhat unfamiliar to an English-speaking audience. N/A=not assessed.

## Discussion

We designed an EBHC course that integrated the assessment of media health claims, along with the use of a blended learning approach and a group exam. Our study aimed to explore healthcare students’ experiences with this course. To our knowledge, this is the first study to examine the experiences of bachelor healthcare students where the use of health claims for critical thinking is applied. Health claims from the media served as an entry point to EBP and were consistently incorporated into EBP teaching and the exam assignment.

### Main findings

We found that engaging in critical reflection on the reliability of health claims presented in the “Behind the Headlines” module helped introduce healthcare students to the concept of EBP. The implementation of blended learning, which combined online and in-person components, proved beneficial for students’ perceived learning outcomes. In addition, the collaborative exam assignment centered around health claims succeeded not only in promoting critical reflection but also in enhancing students’ perceived learning outcomes. However, the findings are derived from students’ subjective experiences and, therefore, do not allow for causal inferences. The following sections provide a detailed elaboration of our findings.

### Health claims as an entry point for EBP

Our study revealed that health claims, introduced through the “Behind the Headlines” module, served as a helpful entry point for EBP. Five IHC Key Concepts, ^15^ most of which align with the “0.2 Recognize the rationale for EBP” section of Albarqouni et al.’s EBP framework, ^4^ were introduced via this module to foster an understanding of the role of reliable research in informing practical application. Students’ perception of the interrelationship between the “Behind the Headlines” module and EBP was likely influenced by the use of health claims in the initial module, acting as a bridge to the broader context of the EBP course. ^28^

Our results further indicate that students found health claims advantageous for multiple purposes. Rather than starting directly with scientific articles, they found this approach useful for facilitating critical evaluation of health claims in everyday life and preparing for their future roles as healthcare professionals. The ability to critically assess health claims, an aspect of critical health literacy, ^28^^,^^29^ plays a role in protecting individuals against misleading treatment claims and enabling them to make informed decisions regarding their health. ^15^ With the growing prevalence of health claims in various media channels ^30^ and an emphasis on shared decision-making, ^31^^,^^32^ future healthcare professionals are likely to encounter health claims from patients sourced from the media. ^11^ However, the ability to assess health claims remains limited among various individuals, including healthcare professionals. ^33^^,^^34^ Thus, interventions aimed at promoting critical thinking in this domain hold value. Several initiatives, including the strategy described in our current study, as well as other relevant approaches, ^12^^,^^13^^,^^35^^,^^36^ have the potential to facilitate this process. We believe that the integration of health claims into EBP teaching will empower future healthcare professionals to skillfully navigate the vast media landscape. This empowerment may enable healthcare professionals to assist patients in making informed decisions about their health through shared decision-making. Consequently, this approach can result in improved health outcomes, enhanced quality of life, and better access to suitable and cost-effective treatments, ^37^ making it a valuable contribution to evidence-based healthcare.

### The blended learning design for perceived learning outcomes

Our EBHC course employed a blended learning strategy ^38^ with elements from a flipped classroom approach. ^39^ This approach involved utilizing digital learning resources for pre-class preparation and in-class sessions, which included lectures and collaborative problem-solving activities. We found that students appreciated the extensive digital learning resources available both before and after the lectures. Furthermore, the students expressed their appreciation for the wide range of learning activities incorporated during the lectures, such as quizzes, seminars, and peer assessments. For instance, 73.5% of the students agreed that they had experienced positive learning outcomes from problem-solving seminars with teachers. This finding is consistent with the findings of Bala et al.’s systematic review, which emphasized the importance of interactive and multifaceted approaches in EBP education. ^5^ However, students’ experience with breakout rooms revealed that many students were unprepared, leading to frustration during lecture sessions that involved multiple breakout room activities. This finding aligns with findings in a review that identified the drawbacks of a flipped classroom approach, which highlighted the frequent occurrence of insufficient student preparation before in-class sessions. ^40^

Ødegaard et al. ^41^ found, in their systematic review, that various digital learning designs, including blended learning and flipped classrooms, in physiotherapy education demonstrated comparable or superior effects to traditional classroom teaching in terms of knowledge and practical skills acquisition. In addition, Akçayır and Akçayır ^40^ reported that the most common benefit associated with a flipped classroom approach was the enhancement of student learning performance. These findings align with the results of Naing et al., ^42^ who also concluded that the implementation of flipped classrooms has the potential to improve academic achievement and enhance student satisfaction across various health professional programs. Although our study did not specifically assess students’ learning performance in terms of EBP knowledge, skills, and attitudes, most participants (90.5%) reported a positive perception of the overall learning outcome on the Likert scale in the evaluation survey.

Our findings indicate that implementing the active engagement approach through a blended learning design in the EBHC course contributed to a positive perception of the students’ learning achievements. In previous studies, active engagement has consistently demonstrated positive associations with learning outcomes. ^5^^,^^43^ The alignment of our findings with existing research underscores the potential effect of the active engagement approach, as found in blended learning, in promoting favorable learning outcomes for students.

### Group collaboration for critical reflection

A core element of our EBHC course was the collaborative exam assignment, which was designed to promote critical reflection and student engagement, enhance learning outcomes, and assess students’ grasp of EBP principles. The students recognized group collaboration as vital for promoting critical thinking ^44^ and experienced the benefits of collaborative efforts in critically assessing a health claim and discussing the research evidence. Engaging with peers in the group setting probably encouraged them to challenge assumptions, consider alternative viewpoints, and arrive at well-informed conclusions. ^45^

A substantial 78% of participants acknowledged that engaging in exam groups contributed to increased learning outcomes. The FG interviews additionally underscored the students’ recognition of the benefits of group work in enhancing learning outcomes, emphasizing that discussing and learning in a collaborative group setting was beneficial. The interactive exam group discussions and seminars facilitated a collaborative learning environment, encouraging active participation and the exchange of ideas. ^46^ In the open-ended feedback, an emphasis was placed on the advantages of working in groups during the course. This was attributed to the ability to engage in discussions and gain exposure to diverse viewpoints, fostering a deeper understanding of the assignment. Furthermore, feedback from peers was noted to contribute to enhanced insights. This highlights how collaboration among students likely fostered an environment conducive to questioning, seeking clarity, giving constructive feedback, and cultivating a sense of responsibility for meaningful contributions to their group’s advancement. However, the issue of potential free riders emerged as a concern. This aligns with the findings of Donelan and Kear’s systematic review of challenges in online group projects in higher education, which highlighted key issues, including low and uneven student participation. ^47^ Improving the collaborative learning experience can likely be achieved by implementing strategies to solve the free rider problem, such as logging participation in group work, as suggested in the open-ended response. Other strategies for addressing online group project challenges include clear student guidance and preparation, and ongoing practical and emotional support to boost confidence and engagement. ^47^

The incorporation of group collaboration in the course aligns with educational theories that emphasize the value of social interaction in learning. ^48^ Collaborative learning models have been shown to promote critical thinking, problem-solving skills, and communication abilities, ^46^^,^^49^ all of which are integral to EBP. ^5^ The positive outcomes from the collaborative learning experience within the group exam task in our study confirm its relevance and applicability as a strategic component of EBP education and as a useful method for assessing students’ understanding of EBP principles.

### Limitations

Our study has several limitations. Surveys are susceptible to limitations in representing the sample population, such as potential non-response issues. ^50^ In our study, the response rate was relatively low, as we were able to recruit approximately 29% of the students who were enrolled in the EBHC course. This low response rate introduces the possibility of sampling bias, as the characteristics of the respondents may differ systematically from those of the non-responders. ^51^ Consequently, there is an increased risk that the obtained results may not accurately represent the entire study population. ^52^ However, our sample mirrors the general gender distribution of students enrolled in the course, with women making up approximately 78% of the student body compared to approximately 79% in the sample. Moreover, our aim was not specifically focused on attaining statistical generalizability. Instead, we sought to delve into students’ experiences with the EBHC course, using the convergent mixed methods design to enhance our understanding. Adding to this context, 80.1% (109/136) of respondents provided positive feedback about the course, and 72.0% (98/136) provided constructive suggestions for improvement. These response rates demonstrate a high level of engagement and participation from the individuals surveyed.

Participants from the various courses were included in the FG interviews, ensuring representation across the board. Nevertheless, the presence of lecturers as both moderators and note-takers during the interviews may have introduced social desirability bias, ^53^ leading participants to provide responses that align with researchers’ expectations. It is noteworthy, however, that the findings derived from the FG interviews were consistent with the anonymous responses obtained through open-ended questions in the evaluation survey. This alignment of results suggested that the responses were not susceptible to the influence of social desirability bias.

The primary objective of this study was to assess the experiences of healthcare students in the EBHC course, with a specific emphasis on the integration of health claims, student-active learning activities and a group exam assignment. However, this study is part of a broader feasibility project. ^54^ Therefore, some findings that are not included in this paper, will contribute to the refinement of the course structure, ultimately facilitating a comprehensive evaluation of students’ learning outcomes using objective measurements. 

## Conclusion

Incorporating critical reflection on media health claims into EBP education, along with a blended learning approach and a group exam, showed promise as a useful approach for educating bachelor healthcare students in this study. However, the only basis for our evaluation was the subjective experiences of the students. Therefore, further research employing more robust study designs will be necessary to objectively assess the effect of our pedagogical design on learning outcomes.

## Data Availability

Zenodo: Supplements and underlying data for the study: Using Health Claims to Teach Evidence-Based Practice to Healthcare Students: A Mixed Methods Study, https://doi.org/10.5281/zenodo.10532808. ^22^ This project contains the following underlying data:
• Quantitative data. 2_All quantitative data_English translation.xlsx • Qualitative data (open-ended questions). 3_All open questions_English translation.docx • Qualitative data (FG interviews). 4_All FG interviews_English translation.docx Zenodo: Supplements and underlying data for the study: Using Health Claims to Teach Evidence-Based Practice to Healthcare Students: A Mixed Methods Study, https://doi.org/10.5281/zenodo.10532808. ^22^ This project contains the following extended data:
• 1_Supplements_.docx •
STROBE_checklist_BHinEBP.docx • Consolidated criteria for reporting qualitative studies.docx • Mixed Methods Approach checklist.docx Data are available under the terms of the Creative Commons Attribution 4.0 International license (CC-BY 4.0).

## References

[1] Lehane E : Evidence-based practice education for healthcare professions: an expert view. *BMJ Evid. Based Med.* 2019;24(3):103–108. 10.1136/bmjebm-2018-111019 30442711 PMC6582731

[2] Sackett DL : Evidence based medicine: what it is and what it isn’t. *BMJ.* 1996;312(7023):71–72. 10.1136/bmj.312.7023.71 8555924 PMC2349778

[3] Dawes M : Sicily statement on evidence-based practice. *BMC Med. Educ.* 2005;5(1):1. 10.1186/1472-6920-5-1 15634359 PMC544887

[4] Albarqouni L : Core Competencies in Evidence-Based Practice for Health Professionals: Consensus Statement Based on a Systematic Review and Delphi Survey. *JAMA Netw. Open.* 2018;1(2):e180281. 10.1001/jamanetworkopen.2018.0281 30646073

[5] Bala MM : What are the effects of teaching Evidence-Based Health Care (EBHC) at different levels of health professions education? An updated overview of systematic reviews. *PLoS One.* 2021;16(7):e0254191. 10.1371/journal.pone.0254191 34292986 PMC8297776

[6] Ministry of Education and Research: *National Curriculum Regulations for Norwegian Health and Welfare Education [Forskrift om felles rammeplan for helse- og sosialfagutdanninger], in FOR-2017-09-06-1353.* Lovdata;2017.

[7] Guyatt G : Evidence-Based Medicine: A New Approach to Teaching the Practice of Medicine. *JAMA.* 1992;268(17):2420–2425. 10.1001/jama.1992.03490170092032 1404801

[8] Aglen B : Pedagogical strategies to teach bachelor students evidence-based practice: A systematic review. *Nurse Educ. Today.* 2016;36:255–263. 10.1016/j.nedt.2015.08.025 26375570

[9] Oxman M : Using claims in the media to teach essential concepts for evidence-based healthcare. *BMJ Evid. Based Med.* 2021;26(5):234–236. 10.1136/bmjebm-2020-111390 33158855 PMC8479747

[10] Boutron I : Three randomized controlled trials evaluating the impact of “spin” in health news stories reporting studies of pharmacologic treatments on patients’/caregivers’ interpretation of treatment benefit. *BMC Med.* 2019;17(1):105. 10.1186/s12916-019-1330-9 31159786 PMC6547451

[11] Bekkum JE van Hilton S : Primary care nurses’ experiences of how the mass media influence frontline healthcare in the UK. *BMC Fam. Pract.* 2013;14:178. 10.1186/1471-2296-14-178 24267614 PMC4222829

[12] Nsangi A : Effects of the Informed Health Choices primary school intervention on the ability of children in Uganda to assess the reliability of claims about treatment effects: a cluster-randomised controlled trial. *Lancet.* 2017;390(10092):374–388. 10.1016/S0140-6736(17)31226-6 28539194

[13] Semakula D : Effects of the Informed Health Choices podcast on the ability of parents of primary school children in Uganda to assess claims about treatment effects: a randomised controlled trial. *Lancet.* 2017;390(10092):389–398. 10.1016/S0140-6736(17)31225-4 28539196

[14] Tuntland H Nordheim L : Teaching and learning in evidence-based practice. Presentation of the CASP-model [Undervisning og læring i kunnskapsbasert praksis. Presentasjon av CASP-modellen]. *Ergoterapauten.* 2009:9.

[15] Oxman AD Chalmers I Dahlgren A : Key concepts for informed health choices: Where’s the evidence? [version 2; peer review: 3 approved]. *F1000Research.* 2023;11:890. 10.12688/f1000research.123051.2 37928808 PMC10623542

[16] Creswell JW Creswell JD : *Research design: Qualitative, Quantitative & Mixed Methods Approaches.* 5th ed. SAGE Publications;2018.

[17] Guetterman TC Fetters MD Creswell JW : Integrating Quantitative and Qualitative Results in Health Science Mixed Methods Research Through Joint Displays. *Ann. Fam. Med.* 2015;13(6):554–561. 10.1370/afm.1865 26553895 PMC4639381

[18] Elm E von : The Strengthening the Reporting of Observational Studies in Epidemiology (STROBE) statement: guidelines for reporting observational studies. *PLoS Med.* 2007;4(10):e296. 10.1371/journal.pmed.0040296 17941714 PMC2020495

[19] Tong A Sainsbury P Craig J : Consolidated criteria for reporting qualitative research (COREQ): a 32-item checklist for interviews and focus groups. *Int. J. Qual. Health Care.* 2007;19(6):349–357. 10.1093/intqhc/mzm042 17872937

[20] Hong QN : Mixed methods appraisal tool (MMAT), version 2018. *Registration of copyright.* 2018;1148552(10).

[21] World Medical Association: World Medical Association Declaration of Helsinki: Ethical Principles for Medical Research Involving Human Subjects. *JAMA.* 2013;310(20):2191–2194. 10.1001/jama.2013.281053 24141714

[22] Elvsaas I-KO : Supplements and underlying data for the study: Using Health Claims to Teach Evidence-Based Practice to Healthcare Students: A Mixed Methods Study. *Zenodo.* 2024. 10.12688/f1000research.146648.3 PMC11602695 39610403

[23] IBM Corp: *IBM SPSS Statistics for Windows.* Armonk, NY: IBM Corp;2020.

[24] Malterud K : Systematic text condensation: A strategy for qualitative analysis. *Scand. J. Public Health.* 2012;40(8):795–805. 10.1177/1403494812465030 23221918

[25] Rosenbaum S : Improving the user experience of evidence: a design approach to evidence-informed health care. Oslo: Oslo School of Architecture and Design;2010.

[26] Dodgson JE : Reflexivity in Qualitative Research. *J. Hum. Lact.* 2019;35(2):220–222. 10.1177/0890334419830990 30849272

[27] Graneheim UH Lundman B : Qualitative content analysis in nursing research: concepts, procedures and measures to achieve trustworthiness. *Nurse Educ. Today.* 2004;24(2):105–112. 10.1016/j.nedt.2003.10.001 14769454

[28] Oxman AD García LM : Comparison of the Informed Health Choices Key Concepts Framework to other frameworks relevant to teaching and learning how to think critically about health claims and choices: a systematic review. *F1000Res.* 2020;9:164–164. 10.12688/f1000research.21858.1 33224475 PMC7670481

[29] Chinn D : Critical health literacy: a review and critical analysis. *Soc. Sci. Med.* 2011;73(1):60–67. 10.1016/j.socscimed.2011.04.004 21640456

[30] Suarez-Lledo V Alvarez-Galvez J : Prevalence of Health Misinformation on Social Media: Systematic Review. *J. Med. Internet Res.* 2021;23(1):e17187. 10.2196/17187 33470931 PMC7857950

[31] Lancet T : Taking shared decision making more seriously. *Lancet.* 2011;377(9768):784. 10.1016/S0140-6736(11)60290-0 21377556

[32] Hoffmann TC Montori VM Del Mar C : The Connection Between Evidence-Based Medicine and Shared Decision Making. *JAMA.* 2014;312(13):1295–1296. 10.1001/jama.2014.10186 25268434

[33] Austvoll-Dahlgren A : The Norwegian public’s ability to assess treatment claims: results of a cross-sectional study of critical health literacy [version 2; peer review: 1 approved, 1 approved with reservations]. *F1000Res.* 2021;9(179). 10.12688/f1000research.21902.2 PMC10995534 38585673

[34] Oxman A : Understanding of key concepts relevant to assessing claims about treatment effects: a survey of Norwegian adults, in IHC Working Paper. Oslo:2017.

[35] Nsangi A : Effects of the Informed Health Choices primary school intervention on the ability of children in Uganda to assess the reliability of claims about treatment effects, 1-year follow-up: a cluster-randomised trial. *Trials.* 2020;21(1):27. 10.1186/s13063-019-3960-9 31907013 PMC6945419

[36] Elvsaas I-KO : Development and Evaluation of a Serious Game Application to Engage University Students in Critical Thinking About Health Claims: Mixed Methods Study. *JMIR Form. Res.* 2023;7:e44831. 10.2196/44831 37166972 PMC10214114

[37] Vahdat S : Patient involvement in health care decision making: a review. *Iran Red. Crescent Med. J.* 2014;16(1):e12454. 10.5812/ircmj.12454 24719703 PMC3964421

[38] Boelens R : Blended learning in adult education: towards a definition of blended learning. 2015.

[39] Abeysekera L Dawson P : Motivation and cognitive load in the flipped classroom: definition, rationale and a call for research. *High. Educ. Res. Dev.* 2015;34(1):1–14. 10.1080/07294360.2014.934336

[40] Akçayır G Akçayır M : The flipped classroom: A review of its advantages and challenges. *Comput. Educ.* 2018;126:334–345. 10.1016/j.compedu.2018.07.021

[41] Ødegaard NB : Digital learning designs in physiotherapy education: a systematic review and meta-analysis. *BMC Med. Educ.* 2021;21(1):48. 10.1186/s12909-020-02483-w 33441140 PMC7805166

[42] Naing C : The effects of flipped classrooms to improve learning outcomes in undergraduate health professional education: A systematic review. *Campbell Syst. Rev.* 2023;19(3):e1339. 10.1002/cl2.1339 37425620 PMC10326838

[43] Prince M : Does Active Learning Work? A Review of the Research. *J. Eng. Educ.* 2004;93(3):223–231. 10.1002/j.2168-9830.2004.tb00809.x

[44] Lai ER : Critical thinking: A literature review. *Pearson’s Research Reports.* 2011;6(1):40–41.

[45] Gokhale AA : Collaborative learning enhances critical thinking. (fall 1995), 1995;7(1).

[46] Laal M Laal M : Collaborative learning: what is it? *Procedia. Soc. Behav. Sci.* 2012;31:491–495. 10.1016/j.sbspro.2011.12.092

[47] Donelan H Kear K : Online group projects in higher education: persistent challenges and implications for practice. *J. Comput. High. Educ.* 2023;1–34. 10.1007/s12528-023-09360-7 PMC10038701 37359044

[48] Okita SY : *Social Interactions and Learning, in Encyclopedia of the Sciences of Learning.* Seel NM , editor. Boston, MA: Springer US;2012; pp.3104–3107.

[49] Voogt J Roblin NP : A comparative analysis of international frameworks for 21st century competences: Implications for national curriculum policies. *J. Curric. Stud.* 2012;44(3):299–321. 10.1080/00220272.2012.668938

[50] Couper MP : Issues of Representation in eHealth Research (with a Focus on Web Surveys). *Am. J. Prev. Med.* 2007;32(5, Supplement):S83–S89. 10.1016/j.amepre.2007.01.017 17466823

[51] Turk A Heneghan C Nunan D : *Non-response bias.* Catalogue of Bias Collaboration;2019.

[52] Partin MR : The impact of survey nonresponse bias on conclusions drawn from a mammography intervention trial. *J. Clin. Epidemiol.* 2003;56(9):867–873. 10.1016/S0895-4356(03)00061-1 14505772

[53] Grimm P : *Social desirability bias.* Wiley international encyclopedia of marketing;2010.

[54] Elvsaas I : Ability to assess claims about treatment effects among bachelor students in health sciences: a protocol for feasibility testing a course in Evidence-Based Health Care. *Zenodo.* 2022.

