# Supplementary material for: Experiences Using Media Health Claims to Teach Evidence-Based Practice to Healthcare Students: A Mixed Methods Study
Source: F1000Res. 2025 Jan 28;13:224. Originally published 2024 Mar 27. [Version 3] doi: 10.12688/f1000research.146648.3 (PMC11602695; doi:10.12688/f1000research.146648.3)
Supplement: Peer review history

# Reviewer report: Reviewer response for version 3

**DOI:** 10.5256/f1000research.177294.r363094

**Published:** 2025-02-06

**Authors:** Wendy Romney

**Affiliations:** 1 Department of Physical Therapy and Human Movement Science, Sacred Heart University, Fairfield, Connecticut, USA

Thank you for your thorough revisions. I approve this submission.

Is the work clearly and accurately presented and does it cite the current literature?

Partly

If applicable, is the statistical analysis and its interpretation appropriate?

Partly

Are all the source data underlying the results available to ensure full reproducibility?

Yes

Is the study design appropriate and is the work technically sound?

Partly

Are the conclusions drawn adequately supported by the results?

Yes

Are sufficient details of methods and analysis provided to allow replication by others?

Partly

Reviewer Expertise:

physical therapy, evidence based practice, mixed methods

I confirm that I have read this submission and believe that I have an appropriate level of expertise to confirm that it is of an acceptable scientific standard.

# Reviewer report: Reviewer response for version 2

**DOI:** 10.5256/f1000research.171631.r337010

**Published:** 2024-11-28

**Authors:** Sharon Tucker

**Affiliations:** 1 University of Central Florida (Ringgold ID: 6243), Orlando, Florida, USA; 2 The Ohio State University, The Ohio State University, Columbus, Ohio, USA

This revised manuscript addresses the previous reviewers' concerns. I do not find the findings (all subjective) overly robust, however, the unique approach of using media health claims to stimulate the EBP thinking process for health professions' students is innovative and meaningful. I have only a few comments for this revision. 

 1. Low response rate and likely biased, however this is addressed in the limitations. 

 2. Not sure what NOK means. 

 3. What is mentimeter in Figure 2?

 4. I would be clear about formulating an EBP question not research in the methods. 

 5. I also think important to be clear (under exam assignment) that the findings should be more than one research article.

 This is all; overall revisions well done.

Is the work clearly and accurately presented and does it cite the current literature?

Yes

If applicable, is the statistical analysis and its interpretation appropriate?

Yes

Are all the source data underlying the results available to ensure full reproducibility?

Yes

Is the study design appropriate and is the work technically sound?

Yes

Are the conclusions drawn adequately supported by the results?

Yes

Are sufficient details of methods and analysis provided to allow replication by others?

Yes

Reviewer Expertise:

EBP, implementation science, ,intervention research

I confirm that I have read this submission and believe that I have an appropriate level of expertise to confirm that it is of an acceptable scientific standard.

# Response

**Published:** 2025-01-21

**Authors:** Ida-Kristin Orjasaeter Elvsaas

**Affiliations:** Faculty of Health Sciences, Oslo Metropolitan University, Oslo, Oslo, Norway

We appreciate Dr. Sharon Tucker’s thoughtful comments on our article about students' experiences with our EBHC course. Your feedback has been carefully considered, and the following responses address your comments. We hope that the revisions and clarifications will meet your expectations.

***1. Low response rate and likely biased, however this is addressed in the limitations.***

*Response from the authors:*

 We appreciate Dr. Sharon Tucker's observation that we addressed the likelihood of a biased sample in the first revision of the article.

***2. Not sure what NOK means.***

*Response from the authors:*

 NOK means Norwegian kroner. However, for ease of understanding, we have converted the value to the approximate US dollar equivalent in the revised article.

***3. What is mentimeter in Figure 2?***

*Response from the authors:*

 Mentimeter ( https://www.mentimeter.com/) is a response tool that allows teachers to interact with students in real-time. We have defined Mentimeter and provided the reference in the revised article.

***4. I would be clear about formulating an EBP question not research in the methods.***

*Response from the authors:*

 Based on this input, as well as Dr. Anice George's comment regarding the necessity of having clear objectives for each strand of the research design, we have included the research questions in the objectives section and a description of the methods used to answer these research questions in the methods section of the revised article.

***5. I also think important to be clear (under exam assignment) that the findings should be more than one research article.***

*Response from the authors:*

 We fully agree that using results from individual studies is inconsistent with the fundamental principles of EBP. Students are trained throughout the EBHC course to use findings from systematic reviews in their decision-making, both for their own practice and in interactions with patients. In the exam task, students had to answer their PICO using a systematic review, a chapter in a clinical decision tool, or a guideline. This has been clarified in the updated article.

# Reviewer report: Reviewer response for version 2

**DOI:** 10.5256/f1000research.171631.r337008

**Published:** 2024-11-27

**Authors:** Anice George

**Affiliations:** 1 Manipal Academy of Higher Education, Manipal, India

This article is written with clarity  and explains the experiences of Health Care students, both quantitatively and qualitatively about media health claims to learn evidence-based practice. The teaching used is a blended approach incorporating interactive teaching and critical thinking process. This research is very relevant in inculcating the concept of EBP in carrying out the care responsibilities by the health care students to provide safe and quality care. The article has given explanation about the Evidence Based Practice in Health Care (EBHC) which was a three weeks course. The study participants were from Bio Engineering, Physiotherapy, Social Education and Occupational therapy. The study design was convergent mixed research design. The ethical considerations and process of data collection are explained by the authors. The EBP teaching steps included, Reflect, Ask, Acquire, Appraise, Apply and Evaluate. Also, participants were asked to critically reflect on the health claims made by media. Critical thinking is an essential ability required for health care professionals.

 Collected data were analyzed collectively across all the four disciplines. Quantitative data were collected through a questionnaire and qualitative data were collected through FGDs. The researchers collected positive feedback about the course from the participants. The major outcomes were that critical reflection on health claims was a useful entry point for EBP the blended learning design had a positive influence on the students’ perceived learning outcomes and that the collaborative group exam assignment promoted both critical reflection and positively impacted perceived learning outcomes.

**Strengths of the study**

- The EBHC course and its implementation along with blended learning, critical thinking is very good.
- The Collaborative group exam is a new method included in the study.
- The data collection process is explained well.

**Weakness **

- The research approach is mentioned as convergent mixed methods. Since there is a quantitative and qualitative strand for the design, it requires clear objective for each of the strands separately and then for the mixed integrative aspect also. On page 13, under the title of ‘Results from the mixed methods synthesis’, the researchers have clearly given each of the strands evaluated what, as well as the main outcomes.  Hence it is expected to give the clear objectives which will give more clarity for the mixed methods research approach utilized by the researchers. (Major)
- The participants for the qualitative phase are taken by convenience, instead some criteria-based selection would have been better.
- The FG group discussion audio recording would have been much better to validate the data.

**Suggestions**

- Apart from the above comments the following also may be noted and rectified:

1. Under the heading ‘Objective” the sentence starts like ‘The study aimed to explore” it may be given as an objective statement.
2. The first two sentences under “The EBHC Course” (Page 5) are repetitions. This may be taken care of.

**Relevance**

 This study is quite relevant for the health care profession, as EBP is essential to ensure high quality safe health care services. The critical thinking and blended learning approach is very good in teaching EBP.

Is the work clearly and accurately presented and does it cite the current literature?

Yes

If applicable, is the statistical analysis and its interpretation appropriate?

Yes

Are all the source data underlying the results available to ensure full reproducibility?

Yes

Is the study design appropriate and is the work technically sound?

Yes

Are the conclusions drawn adequately supported by the results?

Yes

Are sufficient details of methods and analysis provided to allow replication by others?

Yes

Reviewer Expertise:

Adolescent Health, Migraine, Hypertension, Child safety and IPE

I confirm that I have read this submission and believe that I have an appropriate level of expertise to confirm that it is of an acceptable scientific standard, however I have significant reservations, as outlined above.

# Response

**Published:** 2025-01-21

**Authors:** Ida-Kristin Orjasaeter Elvsaas

**Affiliations:** Faculty of Health Sciences, Oslo Metropolitan University, Oslo, Oslo, Norway

We greatly appreciate Dr. Anice George’s insightful feedback on our article regarding students' experiences with our EBHC course. Your feedback has been carefully considered, and the following responses address your comments. We hope that the revisions and clarifications will meet your expectations.

***The research approach is mentioned as convergent mixed methods. Since there is a quantitative and qualitative strand for the design, it requires clear objective for each of the strands separately and then for the mixed integrative aspect also. On page 13, under the title of ‘Results from the mixed methods synthesis’, the researchers have clearly given each of the strands evaluated what, as well as the main outcomes.  Hence it is expected to give the clear objectives which will give more clarity for the mixed methods research approach utilized by the researchers. (Major)***

*Response from the authors:*

 We recognize that the research design requires objectives for both the quantitative and qualitative components, as well as a rationale for integrating the datasets. We have revised the article accordingly.

***The participants for the qualitative phase are taken by convenience, instead some criteria-based selection would have been better.***

*Response from the authors:*

 The eligibility criteria for participation in the focus group interviews was that students had to have completed the EBHC course. In the revised article, we have rephrased the relevant sentence to include this.

***The FG group discussion audio recording would have been much better to validate the data.***

*Response from the authors:*

 We fully agree that the best course of action would have been to audio-record the focus group interviews. However, this was not feasible in our study. Instead, two note-takers took individual notes from the discussions. To ensure equal understanding of the discussions, the notes were compared and discussed by both the note-takers and the moderator immediately after the focus group interviews. Any discrepancies were resolved by consensus. Given the circumstances, this was the only way we could ensure the validity of the notes in this study.

**Suggestions**

 Apart from the above comments the following also may be noted and rectified:

***Under the heading ‘Objective” the sentence starts like ‘The study aimed to explore” it may be given as an objective statement.***

*Response from the authors:*

 In response to the suggestion, we have rephrased this sentence in the revised article.

***The first two sentences under “The EBHC Course” (Page 5) are repetitions. This may be taken care of.***

*Response from the authors:*

 We appreciate the observation, and the repeated sentence has been removed from the revised article.

# Reviewer report: Reviewer response for version 2

**DOI:** 10.5256/f1000research.171631.r324328

**Published:** 2024-10-07

**Authors:** Conor Gilligan

**Affiliations:** 1 Bond University, Robina, Queensland, Australia

I am happy to accept the responses made by the authors and appreciate their comprehensive consideration of each point.

Is the work clearly and accurately presented and does it cite the current literature?

Yes

If applicable, is the statistical analysis and its interpretation appropriate?

Partly

Are all the source data underlying the results available to ensure full reproducibility?

Yes

Is the study design appropriate and is the work technically sound?

Yes

Are the conclusions drawn adequately supported by the results?

Yes

Are sufficient details of methods and analysis provided to allow replication by others?

Partly

Reviewer Expertise:

Medical Education, Public Health, Social Sciences, Healthcare Communication

I confirm that I have read this submission and believe that I have an appropriate level of expertise to confirm that it is of an acceptable scientific standard.

# Reviewer report: Reviewer response for version 1

**DOI:** 10.5256/f1000research.160754.r285772

**Published:** 2024-07-04

**Authors:** Conor Gilligan

**Affiliations:** 1 Bond University, Robina, Queensland, Australia

The paper is well written and clear. The limitation of the evaluation to very low-level outcomes (student perceptions) needs to be acknowledged.

 The paper is set up (though title and abstract) as focussing on the health claims aspect but in fact the evaluation and results focus more on the course as a whole including the groupwork approaches and learning management system. The authors could focus the presented results and discussion more on the health claims aspect to make the paper more concise and aligned with their research question.

 Please also consider:

- The demographics of participants is provided but to what extent are participants representative of those enrolled in the course?

Is the work clearly and accurately presented and does it cite the current literature?

Yes

If applicable, is the statistical analysis and its interpretation appropriate?

Partly

Are all the source data underlying the results available to ensure full reproducibility?

Yes

Is the study design appropriate and is the work technically sound?

Yes

Are the conclusions drawn adequately supported by the results?

Yes

Are sufficient details of methods and analysis provided to allow replication by others?

Partly

Reviewer Expertise:

Medical Education, Public Health, Social Sciences, Healthcare Communication

I confirm that I have read this submission and believe that I have an appropriate level of expertise to confirm that it is of an acceptable scientific standard, however I have significant reservations, as outlined above.

# Response

**Published:** 2024-09-08

**Authors:** Ida-Kristin Orjasaeter Elvsaas

**Affiliations:** Faculty of Health Sciences, Oslo Metropolitan University, Oslo, Oslo, Norway

Dear Dr. Gilligan,

 We appreciate the insightful feedback on our article about students' experiences with our EBHC course. We have carefully considered your feedback and addressed your comments in the responses that follow. We hope that the revisions and clarifications will meet your expectations.

**Reviewers feedback**: The limitation of the evaluation to very low-level outcomes (student perceptions) needs to be acknowledged.

**Response**: We have revised the title, abstract, and main body of the article to reflect that this study reports student experiences with our course and that no causal inference can be drawn based on our findings.

**Reviewers feedback**: The paper is set up (though title and abstract) as focussing on the health claims aspect but in fact the evaluation and results focus more on the course as a whole including the groupwork approaches and learning management system. The authors could focus the presented results and discussion more on the health claims aspect to make the paper more concise and aligned with their research question.

**Response**: We recognize that our article is comprehensive and could have potentially been condensed. However, media health claims are intricately incorporated throughout the course. We regret our inability to explicitly state that health claims formed a crucial part of our learning design. The article has been revised to indicate that this study evaluated the full EBHC course, wherein health claims were consistently used to achieve learning outcomes through both the learning activities and the group exam assignment.

**Reviewers feedback: **Please also consider:

- The demographics of participants is provided but to what extent are participants representative of those enrolled in the course?

**Response**: Most of the study participants were women, constituting 79.4% of the sample. The included sample broadly mirrors the overall gender distribution of students enrolled in the EBHC course, with women constituting about 78% of the enrolled student body. The Limitations section of the article has been updated with this information.

 Once again, we would like to express our gratitude for your constructive feedback and valuable suggestions, which have helped to improve the quality of our article.

# Reviewer report: Reviewer response for version 1

**DOI:** 10.5256/f1000research.160754.r260812

**Published:** 2024-04-26

**Authors:** Wendy Romney

**Affiliations:** 1 Department of Physical Therapy and Human Movement Science, Sacred Heart University, Fairfield, Connecticut, USA

This article highlights a novel teaching approach to evidence based practice using media health claims.  It uses mixed methods to evaluate the teaching approach from a variety of students who completed the course.  It is well written and clear.

 In terms of citations: consider other EBP research in physiotherapy:

 1. Kaplan S et al, 2016 [Ref 1]

 2.Chen Y et al, 2023 [Ref 2]

 Minor recommendations:

 Consider naming, add “media” health claims in the title and define it by providing an example right away.   

 Provide a “behind the headlines” example so it is clear what health claims means.  The reader is left to guess what this could mean until they click on a link.  I thought about insurance claims.  A worked example would also provide the reader with the ability to reproduce the course.  Please also describe the course.  3 weeks, how many hours? Was the course split between blending learning and flipped classroom?  A general outline would be helpful.

 Is it possible to compare the sample that consented to the sex of students in each of the classes?  Also compare the sizes of the program in general to the response rate?  For example, of the 446 eligible, what % were from each program?  Was the response rate comparable across program types?   

 Table 1. Please provide all 5 of the IHC Key Concepts that were used versus just 3.   The reader is left to click on the link to find the 49 key concept.

 Under data collection:  please describe 'the learning outcomes were further explored through eight questions'- what are these eight questions?  Suggestion summarizing eight questions as ‘other learning strategies used’ or refer to table 3.  

 Methods:  Is it possible to see if there were any differences in quantitative data between program type? Separate courses were conducted for each program and focus groups were conducted by program type, yet the quantitative analysis isn’t separated.  Even though the shell was the same, some course instructors may have been different in delivery and could explain some of the findings. Or not, but then the discussion could include information on that as support for the flipped classroom style.

 Please write out IHC the first time and describe the IHC key concept framework for those who are not familiar with it.  It appears to just be published. How were 5 key concepts were selected from the 49? Are the 5 key concepts the lower rows on table 1? Details of this are needed.  Please update citation on the IHC key concepts: https://f1000research.com/articles/11-890

Is the work clearly and accurately presented and does it cite the current literature?

Partly

If applicable, is the statistical analysis and its interpretation appropriate?

Partly

Are all the source data underlying the results available to ensure full reproducibility?

Yes

Is the study design appropriate and is the work technically sound?

Partly

Are the conclusions drawn adequately supported by the results?

Yes

Are sufficient details of methods and analysis provided to allow replication by others?

Partly

Reviewer Expertise:

Physical Therapy, Evidence Based Practice, Education

I confirm that I have read this submission and believe that I have an appropriate level of expertise to confirm that it is of an acceptable scientific standard, however I have significant reservations, as outlined above.

## References

[1] : Strategies for Using the APTA Section on Research Evidence-Based Practice Curriculum Guidelines. *Journal of Physical Therapy Education* .2016;30(2) : 10.1097/00001416-201630020-00006 23-31 10.1097/00001416-201630020-00006

[2] : Evidence-Based Practice Doctor of Physical Therapy Curricular Guideline Utilization: A Convergent Parallel Mixed Methods Study. *J Phys Ther Educ* .2023;37(1) : 10.1097/JTE.0000000000000256 9-16 10.1097/JTE.0000000000000256 38478837

# Response

**Published:** 2024-09-08

**Authors:** Ida-Kristin Orjasaeter Elvsaas

**Affiliations:** Faculty of Health Sciences, Oslo Metropolitan University, Oslo, Oslo, Norway

Dear Dr. Romney,

 We are grateful for your insightful feedback on our article regarding students' experiences with our EBHC course. We have thoroughly considered your suggestions and addressed your comments in the subsequent responses. We sincerely apologize for the delay in our response and greatly appreciate your patience. We hope that you will find the modifications we have made to the article and the explanations provided below satisfactory.

**Reviewers comment**: In terms of citations: consider other EBP research in physiotherapy:

 1. Kaplan S et al, 2016 [Ref 1]

 2.Chen Y et al, 2023 [Ref 2]

**Response**: We appreciate your input on the literature. However, we find that these references may not be entirely relevant to our study, which encompasses healthcare students more broadly. In the revised version, we have introduced multiple clarifications about our learning design, hoping to shed more light on the structure of our EBHC course.

*Elaboration*: In our enthusiasm to disseminate the students' experiences with our course, we may have inadvertently overlooked the inclusion of sufficient detail about the specific Norwegian context within which EBP is taught. In Norway, we have spent considerable time developing an approach to EBP education based on the Norwegian adaption of the CASP model (detailed (in Norwegian) in: Tuntland H, Nordheim L. Teaching and learning in evidence-based practice. Presentation of the CASP-model, Ergoterapauten. 2009;9), which has been adopted by most Norwegian institutions teaching EBP. Our unique contribution to this learning design involves the consistent integration of health claims, the encouragement of active student learning through a blended learning approach, and the application of a group exam to assess the course's learning outcomes. Our main objective was to explore students' experiences with these added components. We hope that our revisions provide a more detailed understanding of our study. Despite being set in a Norwegian context, we believe this study may capture the interest of those looking to integrate similar elements into their own EBP context.

**Reviewers comment**: Consider naming, add “media” health claims in the title and define it by providing an example right away. Provide a “behind the headlines” example so it is clear what health claims means. The reader is left to guess what this could mean until they click on a link. I thought about insurance claims. A worked example would also provide the reader with the ability to reproduce the course.  Please also describe the course. 3 weeks, how many hours? Was the course split between blending learning and flipped classroom?  A general outline would be helpful.

**Response**: We have updated the title to include the term "media health claims" and have emphasized that this study focuses on students' experiences with our course. We have also added generic examples of health claims in the Background section and real examples from exam assignments in the section about the exam paper.

 Our initial plan was to employ a flipped classroom approach. However, the course implementation leaned towards a broader blended learning approach, which included elements from the flipped classroom approach. We have added a general course description to the article, while details (the course's general timetable) can be found in the extended data file.

**Reviewers comment**: Is it possible to compare the sample that consented to the sex of students in each of the classes?  Also compare the sizes of the program in general to the response rate? For example, of the 446 eligible, what % were from each program?  Was the response rate comparable across program types?   

**Response**: During the revision process, an error was identified in the count of eligible participants for the EBHC course. The corrected total of participants is 465, including 70 eligible bioengineering students, 159 eligible physiotherapy students, 161 eligible social education students, and 75 eligible occupational therapy students. Consequently, the overall response rate, following the correction of the total number of eligible students, is 29.2%. The response rate, relative to the number of students in each study program, was as follows: bioengineering (48.6%), physiotherapy (27.7%), social education (19.9%), and occupational therapy (34.7%). There could be several factors influencing a student's decision to respond to our voluntary evaluation questions. Still, the differences in response rates between the study programs are not deemed relevant to our overall aim with the study. The quantitative portion serves as one of three sources for gaining insights about the general experiences of healthcare students with our course. This point has been emphasized by extending the Limitations section of the article.

**Reviewers comment**: Table 1. Please provide all 5 of the IHC Key Concepts that were used versus just 3. The reader is left to click on the link to find the 49 key concept.

**Response**: A new table that better captures the five key concepts taught in "Behind the Headlines" is included in the revised article.

**Reviewers comment**: Under data collection: please describe 'the learning outcomes were further explored through eight questions'- what are these eight questions? Suggestion summarizing eight questions as ‘other learning strategies used’ or refer to table 3.  

**Response**: We have restructured this sentence to enhance its readability and comprehension.

**Reviewers comment**: Methods: Is it possible to see if there were any differences in quantitative data between program type? Separate courses were conducted for each program and focus groups were conducted by program type, yet the quantitative analysis isn’t separated. Even though the shell was the same, some course instructors may have been different in delivery and could explain some of the findings. Or not, but then the discussion could include information on that as support for the flipped classroom style.

**Response**: The quantitative and qualitative data were independently collected for each study program. However, all datasets were collectively analyzed across disciplines during the analysis phase, separate and in a joint display. Our approach was informed by our primary objective of gathering experiences from students across various healthcare disciplines, rather than investigating the differences between these fields. While the quotations drawn from the qualitative datasets are presented with their associated study specializations, this method was implemented primarily due to its customary application in qualitative research. We endeavored to select quotes from diverse fields of study, underscoring the universality of these perceptions across all disciplines. However, to prevent an emphasis on study specializations, we have now omitted the students' study affiliation associated with their quotes in the revised article.

 Furthermore, the article has been updated with a new section to describe the learning activity structure, which was comparable across disciplines.

**Reviewers comment**: Please write out IHC the first time and describe the IHC key concept framework for those who are not familiar with it. It appears to just be published. How were 5 key concepts were selected from the 49? Are the 5 key concepts the lower rows on table 1? Details of this are needed.  Please update citation on the IHC key concepts: https://f1000research.com/articles/11-890

**Response**: The present study is partly part of a broader "Behind the Headlines" project at OsloMet, which commenced in 2018. During the initial stages of this project, lectures at OsloMet, external researchers, and students collaborated to identify IHC Key Concepts deemed essential for assessing health claims, resulting in the inclusion of five concepts. This initial phase of the project is detailed in Oxman M, Habib L, Jamtvedt G, Kalsnes B, Molin M. Using claims in the media to teach essential concepts for evidence-based healthcare. BMJ Evidence-Based Medicine. 2021;26(5):234-6. We have updated our article with a brief description of the original “Behind the Headlines” learning design, which was implemented in a condensed form in our EBHC course. Additionally, the five key concepts included in the EBHC course are those presented in the lower rows of Table 1. However, in the revised version of the article, we have provided an alternative overview of the key concepts used. The citation is also updated. 

 Once again, we would like to express our gratitude for your insightful comments and valuable suggestions, which have greatly contributed to enhancing the quality of our article.
